# Does the Integration of Haptic and Visual Cues Reduce the Effect of a Biased Visual Reference Frame on the Subjective Head Orientation?

**DOI:** 10.1371/journal.pone.0034380

**Published:** 2012-04-11

**Authors:** Marc Gueguen, Nicolas Vuillerme, Brice Isableu

**Affiliations:** 1 Univ Paris Sud, URCIAMS-Motor Control & Perception team, Orsay, France; 2 AGIM (AGeing, Imagery, Modelling), FRE 3405, CNRS-UJF-UPMF-EPHE, AFIRM Team, Grenoble, France; The University of Western Ontario, Canada

## Abstract

**Background:**

The selection of appropriate frames of reference (FOR) is a key factor in the elaboration of spatial perception and the production of robust interaction with our environment. The extent to which we perceive the head axis orientation (subjective head orientation, SHO) with both accuracy and precision likely contributes to the efficiency of these spatial interactions. A first goal of this study was to investigate the relative contribution of both the visual and egocentric FOR (centre-of-mass) in the SHO processing. A second goal was to investigate humans' ability to process SHO in various sensory response modalities (visual, haptic and visuo-haptic), and the way they modify the reliance to either the visual or egocentric FORs. A third goal was to question whether subjects combined visual and haptic cues optimally to increase SHO certainty and to decrease the FORs disruption effect.

**Methodology/Principal Findings:**

Thirteen subjects were asked to indicate their SHO while the visual and/or egocentric FORs were deviated. Four results emerged from our study. First, visual rod settings to SHO were altered by the tilted visual frame but not by the egocentric FOR alteration, whereas no haptic settings alteration was observed whether due to the egocentric FOR alteration or the tilted visual frame. These results are modulated by individual analysis. Second, visual and egocentric FOR dependency appear to be negatively correlated. Third, the response modality enrichment appears to improve SHO. Fourth, several combination rules of the visuo-haptic cues such as the Maximum Likelihood Estimation (MLE), Winner-Take-All (WTA) or Unweighted Mean (UWM) rule seem to account for SHO improvements. However, the UWM rule seems to best account for the improvement of visuo-haptic estimates, especially in situations with high FOR incongruence. Finally, the data also indicated that FOR reliance resulted from the application of UWM rule. This was observed more particularly, in the visual dependent subject. Conclusions: Taken together, these findings emphasize the importance of identifying individual spatial FOR preferences to assess the efficiency of our interaction with the environment whilst performing spatial tasks.

## Introduction

The selection of appropriate frames of reference (FOR) appears to be a key factor in the elaboration of spatial perception and the production of robust interaction with our environment. The extent to which we perceive, with both accuracy and precision, the orientation of the head axis (subjective head orientation, SHO) likely contributes to the efficiency of these spatial interactions. An accurate perception of spatial orientation is necessary for maintaining balance and judging object orientation in a gravito-inertial field. Our ability to routinely perceive and control our spatial orientation in a gravito-inertial field (GIF) is based on the functional alignment of egocentric reference frame axes [1]–[3] either on GIF directions or on surrogates of gravity direction e.g., axes of the visual FOR (wall, ground, ceiling). Depending on the task-specific inertial-acceleration constraints [2], axes of the body's different coordinate systems (articular geometrical axes [4]–[6] and/or axes related to body mass distribution [1], [7]) can be advantageously exploited, each belonging to distinct frames of reference.

It is well established that our perception of body orientation (SBO) is altered during passive roll body tilt [8]–[10]. Tilting the body by altering SBO usually produces two kinds of errors: for body tilt angles of less than 60 degrees, SBO underestimates the physical angle between the body and gravity direction (i.e., body orientation is perceived as being less tilted than in reality). Beyond 90 degrees of body tilt, SBO overestimates the body-gravity angle (i.e., body orientation is perceived as being more tilted than in reality). The restoration of somato-proprioceptive cues and efference copies during active body tilt improves SBO in comparison with passive body-tilt conditions [11]. Interestingly, recent studies have also shown that when subjects were asked to actively maintain body alignment with the direction of gravitational pull, the deviation of the head-trunk unit centre of mass altered subjective visual vertical (SVV) estimates [1]. This finding suggests that the subjective vertical is partly derived from proprioceptive cues related to body mass distribution variables, which are not aligned with the body Z-axis in numerous postural activities. This assumption has received both indirect [12]–[14] and direct support [15]. It is also well established that the non alignment of visual frame of reference axes (e.g., tilted frame) with respect to the direction of body axes alters both the subjective vertical and postural vertical [16]–[19].

We assumed that SHO errors could provide interesting clues about the computational processes underlying our perceptions of body orientation and more specifically how the various sensory modalities (visual, vestibular, proprioceptive, interoceptive) work together. Multiple sensory cues combined in an optimal way allow producing more reliable and less biased estimates [20]. Recent studies provide indirect evidence that this occurs in SVV tasks [21]. These authors showed that the “Rod and Frame Effect” (RFE) on SVV decreased in the visuo-haptic setting condition (i.e. the rod was adjusted by both holding and seeing it) as compared to visual condition (i.e. the visual rod was adjusted by remote control). This result showed that the amplitude of the frame effect increases with the impoverishment of sensory response modalities. Adding and combining cues from different sensory modalities (e.g., visual and kinaesthetic) reduced the frame effect, but did not cancel it completely [21], [22]. This residual reliance on the visual frame of reference could reflect central processing rules implemented by the nervous system. However, which rules of sensory integration governed the decreasing of the RFE in the visuo-kinaesthetic modality of response observed on SVV remains unknown. Does the combination of visual and kinaesthetic modalities also reduce the centre of mass effect on the SHO?

Using a visual rod alignment task, the present authors addressed these questions by investigating human subjects' ability to perceive the orientation of their head (subjective head orientation) using various response modalities (visual, haptic and visuo-haptic), while confronted to a tilted visual frame of reference and/or deviation of the head centre-of-mass. The issue was that of assessing whether the effects of a tilted visual frame and deviation of the head centre-of-mass on SHO can be disambiguated i.e., reduced by combining multiple cues, and whether the benefit results via implementation of an optimal rule.

An important and unsolved issue concerns the large and systematic inter-individual differences (IDs) which invariably emerged from these tasks [23]–[26]. The origin of these differences remains poorly understood. It is suggested these IDs reflect preferences in the use of frames of reference [3], [25], [27], [28]. However, alternative hypotheses could explain such idiosyncrasies, and would emerge from the way subjects combine cues from the different sensory modalities regardless of the appropriateness (or inappropriateness) of the available frames of reference. Several multisensory integration rules have already been identified. The Winner-Take-All model (WTA) implies that an individual favours the most reliable sensory modality, i.e., the sensory modality with the smallest variance [29]. The perceptual estimates bias in multimodal condition is similar to that obtained in the more reliable sensory modality alone. The Maximum Likehood Estimation rule (MLE) [20] implies that an individual assigns a weight to each sensory modality. This weight is proportionate to their reliability, and further leads to a weighted mean of sensory modalities in a multimodal condition. In both cases, multisensory settings of the rod to head orientation should be biased towards the more reliable sensory modality. The bias can be reduced (or increased) to a greater extent using the WTA rule, whilst the variance of SHO should be optimally reduced by using the MLE rule to fall short of the more reliable modality.

The question raised by the IDs issue in the realm of spatial orientation perception is whether or not these so-called FOR preferences reflect the inability of certain subjects to weight sensory cues in proportion to their reliability, or whether they failed to identify the appropriateness of FOR (the degree of congruence with gravity direction) to optimally combine sensory cues, or, finally, if they weight FOR based on their reliability regardless of the degree of congruence with gravity direction. The aim of this study was also to test whether the use of an optimal rule of sensory integration best accounts for the data obtained by combining visual and haptic cues to produce optimal SHO.

More specifically, we hypothesised that i) the MLE rule should apply in an appropriate manner provided that the FORs available be congruent and unbiased; ii) WTA rule should be more appropriate in a condition where one FOR is biased leading individuals to shift toward the remaining reliable FOR; iii) a simple algebraic unweighted mean (UWM) should appear in a condition of multiple misleading FORs. As a consequence, the multimodal combination should be subtended i) by the MLE rule in the condition of maximum FORs congruence, producing an optimal SHO, in other words a more reliable percept biased in the direction of the more reliable sensory modality, ii) by an unweighted rule of sensory modality combination with severe FORs incongruence balancing the reciprocal influences of sensory modality on both mean bias and variance of the SHO.

With regards to the issue of the well known IDs in the selection of visual and egocentric FORs, we tested whether these idiosyncratic FORs dependencies constrained downstream modes of visual and haptic integration to SHO. Do visual field dependent and visual field independent subjects significantly differ in their use of sensory cue combination rules? Do egocentric field dependent and -independent subjects significantly differ in their use of sensory cue combination rules?

## Methods

### Subjects

Thirteen subjects, aged twenty five years and two months old (±2 years and 2 months), voluntarily took part in the experiment. Written informed consent was sought, as required by the Helsinki declaration and the EA 4042 local Ethics Committee who specifically approved this study. All were right-handed and none presented any history of injury, surgery, or pathology that could affect their ability to perform spatial orientation tests.

### Task and procedures

We investigated the relative contribution of the visual FOR and the egocentric FOR (centre-of-mass) in the processing of SHO. We also investigated the subjects' ability to process SHO in various sensory response modalities (visual, haptic and visuo-haptic), and the subsequent modification of their reliance on either the visual or egocentric FORs.

#### Assessing individual's reliance on the visual FOR: Effect of the tilted frame on SHO

The reliance to the visual FOR was assessed using a high definition TV screen isolated either using 1) a cylinder-shaped optical tunnel; or by 2) an optical 3D rectangular tunnel (similar to the standard 3D RFT, Oltman, 1968) (25° angular size) [30]. The cylindrical optical tunnel was black, 105.5 cm long and 62.5 cm diameter. The optical 3D tunnel (0.6 m long, 30 * 30 cm section) was made of translucent white plastic (3 mm) and was preferred to 2D displays as it is known to produce larger visual frame effect on perceptual estimates [31]. Positioned at the closed end of both types of optical tunnels is a black rod (15° angular size) which can be tilted independently from the frame. Subjects were seated at the end the tunnels and were thus submitted to three visual contexts (no frame vs. frame tilted at 18° to the right vs. frame tilted at 18° to the left). The contribution of cutaneous cues stemming from foot contact with the support surface was limited by asking subjects to straighten their legs in order for their heel to be the sole contact with the ground. The head was unrestrained, but the effect of vestibular cues was minimized by instructing subjects to keep their head upright and as still as possible. Bringoux et al. [21] showed no modulation of the amplitude of the frame effect according to the head maintenance conditions (restrained or unrestrained).

#### Assessing individual's reliance on the egocentric mass FOR: Effect of the deviation of the head centre of mass on SHO

**Figure 1:**
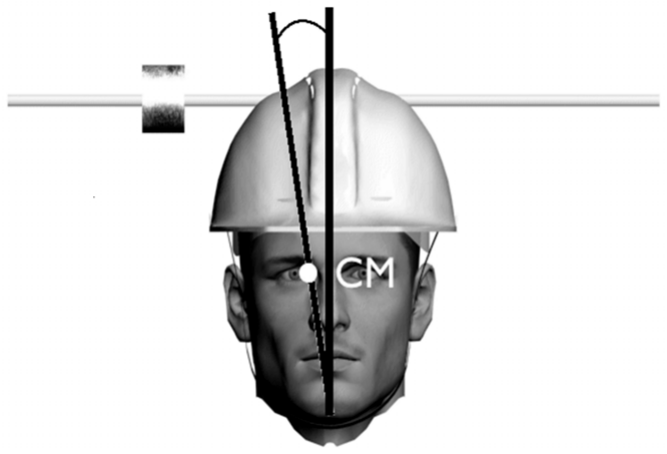
Illustration of the head apparatus which permitted the deviation of head centre of mass.

The centre of mass of the head was deviated by means of a helmet on which masses were added (*Fig. 1*.). The masses were asymmetrically affixed to the side of the head axis at the top of the helmet. A mass of 187 g was placed at an average of 12,77 cm (±2,16 cm) from the centre of the head axis to deviate the head centre of mass by 9,24° (±0,22°). Deviation was coded negatively when shifted towards the subject's left, and positively when shifted towards the right. There were thus three conditions of deviation of the head centre of mass corresponding to −9°, 0°, and +9°. The control condition, corresponding to the deviation of head centre of mass equal to 0°, was obtained without adding masses on the helmet. The deviations of the head centre of mass were computed from the subjects' overall body mass and anthropometric limbs measurements by using regression equations and procedures provided by [32]. Finally, these were adapted from Hanavan's anthropometric geometric model [33].

Subjects were instructed to parallel the rod to the longitudinal axis of their head (i.e. the C7-head vertex direction) across nine conditions: three visual context (no frame vs. frame tilted at 18° to the right vs. frame tilted at 18° to the left) combined with three deviations of the head centre of mass (no deviation vs. deviation to the right vs. deviation to the left). Before each setting, the rod used for response was either tilted at an angle of 18° to the left or to the right. Subjects were instructed to keep their head upright (the head orientation was visually checked by the experimenter). Trials where head leaning was observed were immediately stopped and repeated. A 30 second exploration session allowed subjects to appreciate the modification of the head mass distribution with respect to the body [7]. The sequence in which conditions were imposed was randomized between subjects. Subjects performed four trials per condition (two with the rod initially tilted at 18° to left and two to right).

#### Sensory modalities of response

For each of the nine conditions, subjects were asked to adjust a rod parallel to the perceived longitudinal axis of their head (head z-axis) according to three modalities of response: (1) visual, (2) haptic or (3) visuo-haptic.

**Figure 2:**
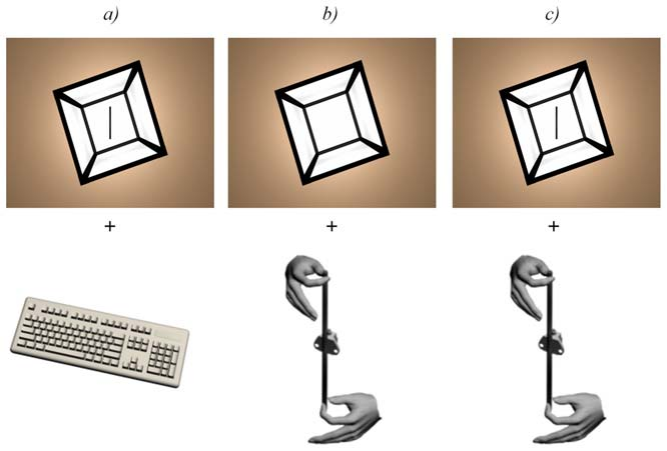
Illustration of frame scene and modes of sensory adjustment: visual (a), haptic (b) and visuo-haptic (c).

1. In the visual modality of response (*Fig. 2a* *.*), the subjects had to adjust a virtual visual rod (of 14° vertical angular size and 0,5° horizontal angular size) displayed on the TV screen, by means of a computer keyboard.
2. In the haptic modality of response (*Fig. 2b*.), the subjects had to adjust a physical rod (measuring 25 cm in length and 1 cm in diameter) held in their hands (between the thumb and the forefinger of each hand, always with the right hand above the left). It must be specified that subjects were not able to see the rod.
3. In the visuo-haptic modality of response (*Fig. 2c* *.*), the displacement of physical rod was associated, in real time, to the same displacement of the virtual rod on the TV screen. The co-alignment of the visual rod with the physical rod was checked before running the experiment.

The haptic and visuo-haptic modalities of response weakly differ in our study from those used by Bringoux et al. [21]. Given that the rod was held and handled with both hands, close to the trunk (about 20 cm), the role of haptic cues was enhanced, whilst upper limb kinaesthetic and biomechanical variables were likely minimized. For this reason, the present authors qualify these sensory conditions of response as haptic and visuo-haptic as opposed to kinaesthetic and visuo-kinaesthetic.

### Data collection and analyses

The orientations of the rod were recorded for each trial with a precision of 0.03°. For both haptic and visuo-haptic modalities of response, final orientations of the rod were recorded using a magnetic sensor, Flock of Birds™ [1]. No time constraint was given to subjects to perform their estimate, who merely had to parallel the rod axis with the head axis as accurately as possible.

We are interested in measuring the precision and accuracy of SHO. Close inspection of our data revealed a main effect of rod starting position, close to significance, in the haptic modality of response (F(1, 12) = 4.127, p = 0.06). In order to cancel out the effect of rod starting position from the whole variance, we first calculated the difference between the mean of trials in each rod starting position condition and the mean of all trials in each condition across rod starting positions. The subsequently obtained value was then subtracted from the value obtained at each trial. The variance and mean of SHO so obtained reflects the effect of FOR perturbation free of rod starting position effect. With these corrected values, we calculated the mean error and variance in each condition and in each modality of response. Finally, to verify whether the decrease of the tilted visual frame effect on SHO in the visuo-haptic modality is due to the use of an optimal rule of combination of visual and haptic cues, we calculated the predicted value in the combined visuo-haptic modality of response, using visual and haptic settings data separately. In the Winner-Take-All model (WTA), the combined estimation (S) of two sources of sensory information and their associated variance is equivalent to the estimation and the variance of the sensory information which has the smallest variance [34].

In the Maximum Likehood Estimation rule (MLE) [20], the combined estimation (*S*) of two sources of sensory information is equivalent to the sum of estimation (*S_i_*) of each sensory information source alone, weighted by the reliability of each cue (*w_i_*).
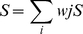
(1)


The reliability (*w_i_*) of each cue is calculated from the variance (*σ_i_*) of each cue.
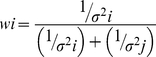
(2)


The variance σ_final_ of the final estimate is
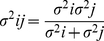
(3)


Final orientations of the rod were subjected to an appropriate analysis of variance to assess the effect of sensory modes of rod adjustments (visual vs haptic vs visuo-haptic) on the amplitude of the visual frame effect as well as the deviation of the head centre of mass on SHO. Only significant (P<.05) results will be reported. ANOVAs and post-hoc tests were performed using STATISTICA 7®.

In order to explore the relationship between visual and egocentric FORs dependencies, and so as to eliminate asymmetric frame and deviation of head CM effect, we applied Nyborg [35] calculation method on our corrected data. So doing, we calculated the constant error of rod settings in each modality of response (visual, haptic and visuo-haptic) in both the tilted visual FOR and deviation of the head's centre of mass conditions. The constant error was calculated by averaging, for each subject, the eight trials performed in each sensory modality of response (visual, haptic and visuo-haptic) in both tilted visual FOR (frame tilted at 18° on the right and on the left) and deviation of the head centre of mass (−9°, 0°, +9°) conditions. Frame effect was then calculated, for each subject, by subtracting the constant error from the mean of the four trials performed when frame tilted to the left (still with each deviation of the head centre of mass) across the three modalities of response. The same procedure was applied in the deviation of the head centre of mass conditions (9° of deviation of the head centre of mass on the right and on the left with each frame condition) to obtain the head centre of mass effect in the visual, haptic and visuo-haptic modalities of response for each subject.

Kolmogorov-Smirnov tests for data normality revealed a normal distribution of errors in each testing condition (351 = 27 conditions ×13 subjects).

## Results

### Effects of the tilted visual FOR and deviation of the head centre of mass on SHO:

**Figure 3:**
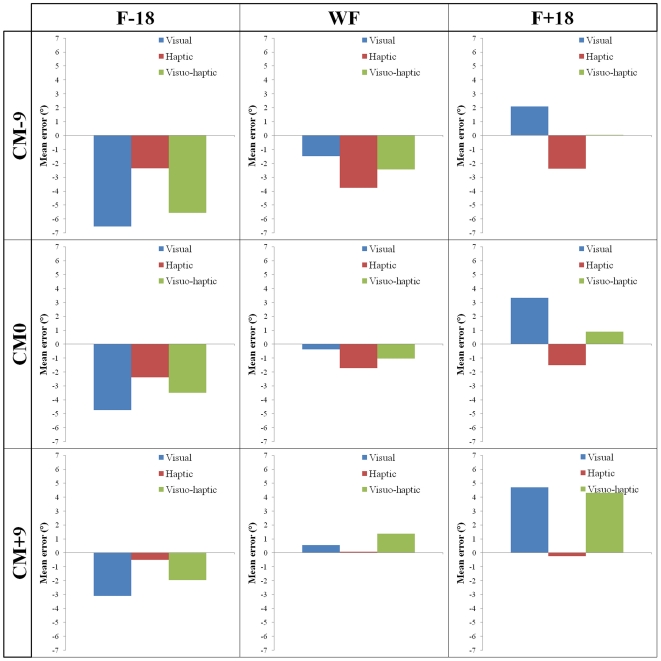
Effect of modalities of response according to frame tilt and deviation of head CM all subjects combined.

The mean SHO across nine conditions (three visual frame orientation * three deviation of head centre of mass) in the visual, haptic and visuo-haptic modalities of response are displayed in *Fig. 3*. A 3×3×3 full-factorial ANOVA (response modality×frame tilt×head CM deviation) carried out on mean SHO error revealed a close to significant main effect for response modality (F(2, 24) = 3.1, p = 0.063), a significant main effect for frame orientation (F(2, 24) = 55.96, p<0.05), but no main effect for head CM deviation. This analysis also revealed a significant interaction effect between response modality and frame tilt (F(4, 48) = 24.49, p<0.05). No interactions effects were reported between response modality and head CM deviation, frame tilt and head CM deviation or between these 3 factors combined.

**Figure 4:**
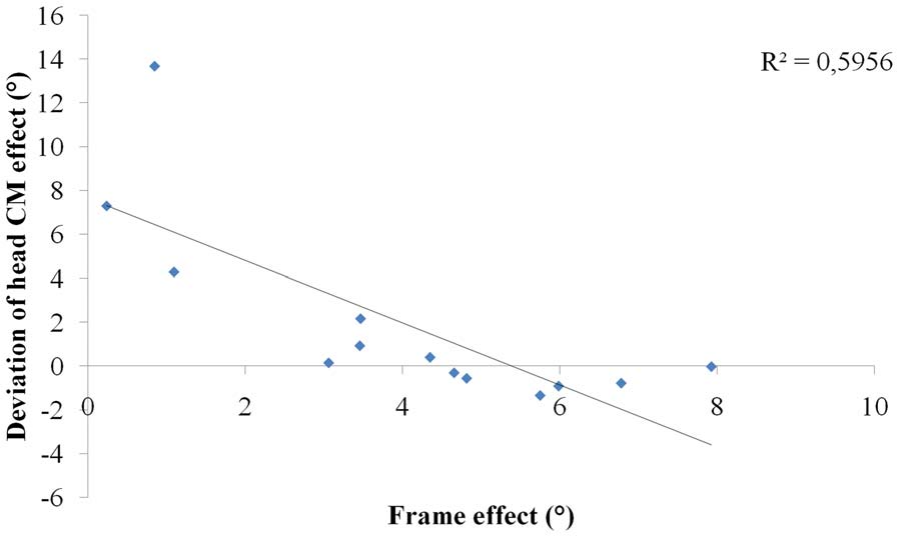
Correlation between individual visual frame effect scores in the visual modality of response in the ‘no deviation of head CM’ condition, and individual head CM effect in the haptic modality of response in the ‘no frame’ condition.

Repeated-measures analyses of variance carried out for each modality of response revealed a significant main effect of frame orientation in the visual (F(2, 24) = 58.17, p<0.05), and visuo-haptic (F(2, 24) = 32.38, p<0.05) modalities of response. No main effect for frame orientation was found in the haptic modality of response. Effect of the head centre of mass deviation was not observed in the visual, haptic and visuo-haptic modalities of response. Repeated measures analyses of variance carried out for each subject and each modality of response revealed a significant main effect of frame orientation for all 13 subjects in the visual modality of response, 3 out of 13 subjects in the haptic modality of response and 11 out of 13 subjects in the visuo-haptic modality of response. The same analysis on an individual level revealed a significant main effect of the deviation of the head centre of mass for 4 out of 13 subjects in the visual modality of response, 2 out of 13 subjects in the haptic modality of response and 8 out of 13 subjects in the visuo-haptic modality of response. The present authors further investigated whether reliance on a FOR (e.g., visual FOR) was inversely correlated with the non use of another FOR (non visual one). To this end, individual visual frame effect scores obtained in the visual modality of response under the ‘no deviation of head CM’ condition (“pure” frame effect), calculated using Nyborg's method, were compared with individual head CM effect in the haptic modality of response under the ‘no frame’ condition (“pure” deviation of head CM effect), also calculated using Nyborg's method. Correlation analysis revealed a significant negative relationship (r = −.77; p<0.05) between visual FOR dependency and egocentric FOR dependency (*Fig. 4*). This would suggest that the more subjects relied on the visual FOR, the less they were influenced by the deviation of the head CM, and conversely.

### Effects of sensory modalities of response on SHO:

The present authors were interested in testing whether the combination of several modalities of response (i.e., visuo-haptic) allowed to reduce the effect of both the tilted visual FOR and head centre of mass deviation on SHO. With this view, we examined responses obtained in the visual and haptic modalities of response separately, and then in the visuo-haptic modality of response for each condition (*Fig. 3*).

A repeated-measures analysis of variance carried out for each condition revealed significant main effects of modality of response when (1) the frame was tilted by +18° combined with deviated head CM at +9° (F(2,24) = 12.48, p<0.05), (2) the frame was tilted at +18° without head CM deviation (F(2,24) = 27.11, p<0.05), (3) the frame was tilted at +18° with deviated head CM at −9° (F(2,24) = 12.33, p<0.05), (4) the frame was tilted at −18° with a head CM deviation of +9° (F(2,24) = 4.7, p<0.05), (5) the frame was tilted at −18° with no head CM deviation (F(2,24) = 3.46, p<0.05) and finally (6) the frame was tilted at −18° with a −9° head CM deviation (F(2,24) = 7.38, p<0.05). No significant modality of response main effect was obtained in (1) the no frame condition when head CM was deviated at +9°, (2) the no frame condition with no head CM deviation, or (3) the no frame condition with a −9° head CM deviation. When the effect of modality of response is significant, the amplitude of the VH mean error is systematically situated between the visual mean error and the haptic mean error. A summary of significant main effects for modality of response across conditions is presented in Table 1.

**Table 1:** Summary of significant main effects modality of response according to frame tilt and deviation of head CM all subjects combined. S = significant effect of response modality, NS = no significant effect of response modality.

	F−18	WF	F+18
**CM−9**	S (F(2, 24) = 7.375; p<0.05)	NS	S (F(2, 24) = 12.333; p<0.05)
**CM0**	S (F(2, 24) = 3.463; p<0.05)	NS	S (F(2, 24) = 27.107; p<0.05)
**CM+9**	S (F(2, 24) = 4.696; p<0.05)	NS	S (F(2, 24) = 12.483; p<0.05)

### Optimal rule of sensory combination (MLE) applied to sensory cues and preferential selection of FOR:

**Figure 5:**
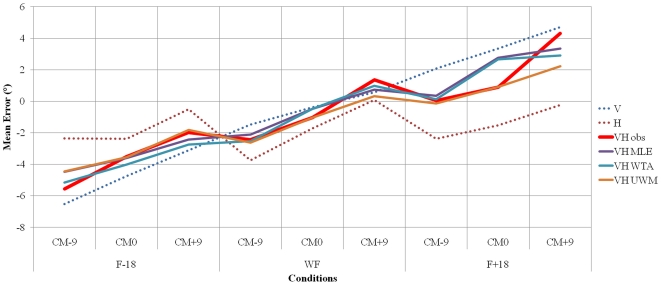
Mean Error according to conditions and modality of response. V = Visual modality of response, H = Haptic modality of response, VH obs = Visuo-haptic modality of response, VH MLE = Visuo-haptic calculated with MLE, VH WTA = Visuo-haptic calculated with WTA, VH UWM = Visuo-haptic calculated with unweighted mean error, F−18 = Frame tilted at 18° to the left, WF = cylinder-shape optical tunnel, F+18 = Frame tilted at 18° to the right, CM−9 = deviation of the head centre of mass at 9° to the left right, CM0 = no deviation of the head centre of mass, CM+9 = deviation of the head centre of mass at 9° to the right.

To check whether the decrease of the tilted visual frame effect on SHO in the visuo-haptic modality is a consequence of the use of an optimal rule of combination of visual and haptic cues, we decided to compute the MLE rule from observed visual and haptic settings data. We applied equations (1), (2) and (3) (see methods) to obtain the predicted visuo-haptic mean error and variance across all nine conditions (*Table 2* *,* *Fig. 5*). The visuo-haptic error predicted by the use of the MLE rule was then compared to the observed visuo-haptic data. Results show that the MLE rule seems to account for the observed visuo-haptic mean bias (no difference between mean error predicted by MLE rule in VH response and mean error observed in VH response). However, this result was not observed with regards to variance (significant difference between variance predicted by MLE rule in VH response and variance observed in VH responses (F(1, 116) = 21.326; p<0.05)); as the variance observed in the visuo-haptic modality of response is always larger than when predicted by the MLE. The present authors hypothesized that SHO should be optimal when using the MLE rule provided that FORs remained unbiased. The same analysis was thus applied in each condition. Results showed that the MLE rule seems account for the observed visuo-haptic mean bias for 8 out of the 9 conditions (i.e. no difference between mean error and variance predicted by MLE rule in VH response; no difference between mean error and variance observed in VH response).

**Table 2:** Mean Error (ME) and Variance (Var) according to conditions and modality of response.

		V		H		VH obs		VH MLE		VH WTA		VH UWM	
		ME	Var	ME	Var	ME	Var	ME	Var	ME	Var	ME	Var
**F−18**	**CM−9**	−6,53	1,46	−2,35	15,42	−5,56	7,61	−4,49	0,67	−5,17	1,08	−4,44	8,44
	**CM0**	−4,74	0,98	−2,39	2,04	−3,51	0,91	−3,61	0,51	−4,00	0,77	−3,56	1,51
	**CM+9**	−3,11	0,69	−0,52	6,53	−1,97	5,09	−2,43	0,48	−2,76	0,61	−1,81	3,61
**WF**	**CM−9**	−1,49	2,14	−3,75	3,06	−2,45	3,88	−2,12	0,98	−2,52	1,52	−2,62	2,60
	**CM0**	−0,37	0,19	−1,72	5,68	−1,03	5,32	−0,49	0,14	−0,47	0,17	−1,05	2,93
	**CM+9**	0,55	0,64	0,09	9,85	1,36	7,74	0,73	0,47	0,97	0,57	0,32	5,25
**F+18**	**CM−9**	2,08	1,79	−2,38	1,87	0,05	9,34	0,33	0,62	0,13	0,90	−0,15	1,83
	**CM0**	3,33	0,75	−1,52	13,86	0,91	9,66	2,76	0,48	2,66	0,57	0,90	7,30
	**CM+9**	4,70	2,02	−0,24	17,22	4,31	7,49	3,34	1,23	2,90	1,97	2,23	9,62

*V = Visual modality of response, H = Haptic modality of response, VH obs = Visuo-haptic modality of response, VH MLE = predicted Visuo-haptic estimate calculated with MLE, VH WTA = predicted Visuo-haptic estimate calculated with WTA, VH UWM = predicted Visuo-haptic estimate calculated with unweighted mean, F−18 = Frame tilted at 18° to the left, WF = cylinder-shape optical tunnel, F+18 = Frame tilted at 18° to the right, CM−9 = deviation of the head centre of mass at 9° to the left right, CM0 = no deviation of the head centre of mass, CM+9 = deviation of the head centre of mass at 9° to the right.*

The observed visuo-haptic data was also compared to the visuo-haptic error predicted by the Winner-Take-All model. Results are similar to those observed with the MLE rule (i.e., no difference between mean error predicted by WTA model in VH response and mean error observed in VH response, but significant difference between variance predicted by WTA model in VH response and variance observed in VH responses (F(1, 116) = 18.776; p<0.05)); as the variance observed in the visuo-haptic modality of response is always larger than that predicted with the WTA. The present authors hypothesized that SHO should result from using the WTA rule of sensory modalities in condition when one FOR is biased. The same analysis was applied in each condition. Results showed that WTA rule seems account for the observed visuo-haptic mean whatever the conditions of FORs orientation (i.e. no difference between mean error and variance predicted by WTA rule in VH response; no difference between mean error and variance observed in VH response).

Ultimately, observed visuo-haptic data was compared to the visuo-haptic error predicted by the algebraic unweighted mean (UWM) of errors and the variance obtained in visual and haptic modalities of response alone. Results suggest that this model accounts for the observed visuo-haptic mean bias (no difference between mean error predicted by this model in VH response and mean error observed in VH response). Furthermore, this rule also accounts for the variance (no significant difference between variance predicted by this model in VH response and variance observed in VH responses). The same analysis was then applied in each condition. Results suggest that the UWM rule accounts for the observed visuo-haptic mean regardless of FORs orientation (i.e. no difference between mean error and variance predicted by UWM rule in VH response; no difference between mean error and variance observed in VH response).

**Figure 6:**
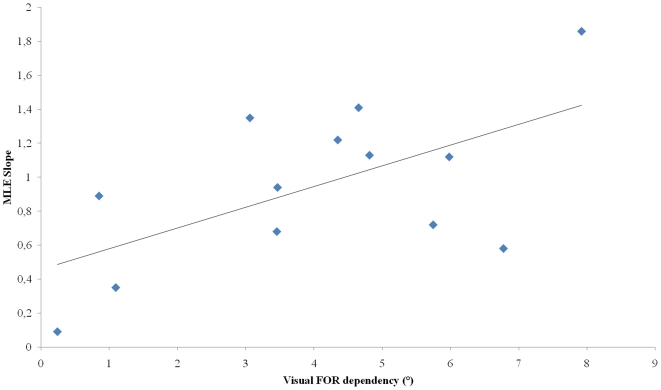
Correlation between individual visual frame effect scores in visual modality of response in the ‘no deviation of head CM’ condition and slope between visuo-haptic response predicted by MLE rule and observed visuo-haptic response.

The present authors also investigated whether the use of the MLE was modulated by the subject's degree of reliance on the visual or non-visual FOR. With this in mind, the slope between visuo-haptic response predicted by MLE rule and visuo-haptic response observed for each subject was calculated. The obtained individual slopes were then used for correlation analysis with visual or egocentric FOR dependency (*Fig. 6*). The analysis showed these slopes to be significantly correlated with visual FOR dependency (R^2^ = 0.36; p<0.05), but not with egocentric FOR dependency. Results thus indicate that the more extreme subjects were (i.e. the heavier their exclusive reliance on the visual or non visual FOR), the more MLE rule overestimated or underestimated the observed VH frame effect on SHO.

**Figure 7:**
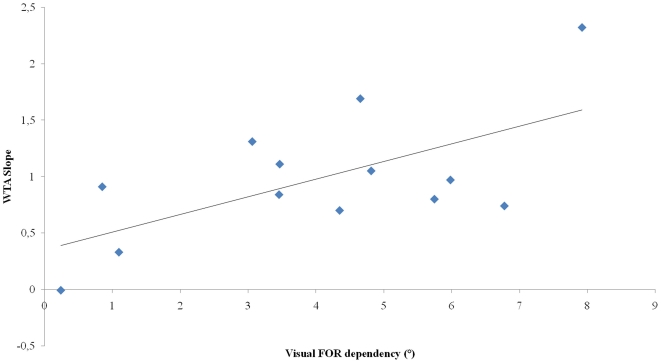
Correlation between individual visual frame effect scores in visual modality of response in the ‘no deviation of head CM’ condition and slope between visuo-haptic response predicted by WTA rule and observed visuo-haptic response.

The same analysis was performed with the WTA rule (*Fig. 7*). Analysis revealed a significant correlation between the slopes and visual FOR dependency (R^2^ = 0.40; p<0.05) but not between the slopes and egocentric FOR dependency. Results show that the more extreme the subjects were (i.e. the heavier their exclusive reliance on visual or non visual FOR), the more WTA rule overestimated or underestimated the observed VH frame effect on SHO.

The same analysis was performed with UWM rule, revealing no significant correlation between the slopes and visual FOR dependency or between the slopes and egocentric FOR dependency.

Taken together, the UWM rule seems to best account for the observed visuo-haptic improvement of estimates. Indeed, this rule accounts for both the mean bias and the variance, whatever FORs orientation. Furthermore, correlation analyses revealed that this rule is independent of the subject's degree of reliance on the visual or non-visual FOR.

### Optimal rule of sensory combination (MLE) applied to FOR

**Figure 8:**
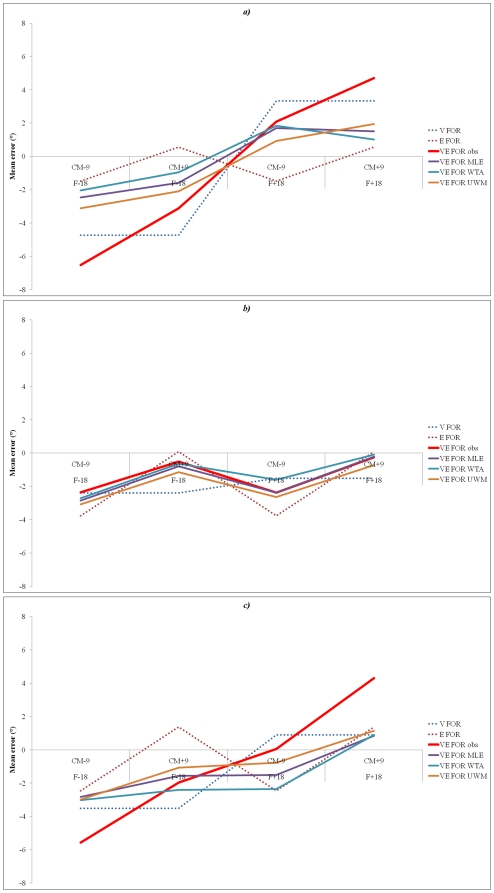
Mean Error according to conditions and modality of response. V FOR = mean error with only visual FOR disrupted (F±18 and CM0), E FOR = mean error with only egocentric FOR disrupted (WF and CM±9), VE FOR obs = mean error observed with visual and egocentric FOR disrupted (F±18 and CM±9), VE FOR MLE = mean error with visual and egocentric FOR disrupted calculated with MLE, VE FOR WTA = mean error with visual and egocentric FOR disrupted calculated with WTA, VE FOR UWM = mean error with visual and egocentric FOR disrupted calculated with unweighted mean error, F−18 = Frame tilted at 18° to the left, WF = cylinder-shape optical tunnel, F+18 = Frame tilted at 18° to the right, CM−9 = deviation of the head centre of mass at 9° to the left, CM0 = no deviation of the head centre of mass, CM+9 = deviation of the head centre of mass at 9° to the right.

Here we sought to identify in what way FORs are combined. We decided to compute the MLE rule from FOR. In other words, we predicted responses in conditions where both FOR were altered (frame tilted combined to deviation of head CM) based on data obtained in conditions with only one altered FOR (frame tilted or head CM deviated). We applied equations (1), (2) and (3) (see methods) to predict data error and variance across all three modalities of response (*Fig. 8*). The predicted mean reliance on FORs resulting from the use of the MLE rule in the condition where both FOR were altered was then compared to the observed mean reliance on FORs in the same condition. Results suggest that the MLE rule accounts for the observed mean reliance on FORs (no difference between mean error predicted by MLE rule and the observed mean reliance on FORs whatever response modality). However, such a result was not observed with regards to variance (significant difference between variance predicted by MLE rule and the observed variance for visual (F(1, 51) = 12.015; p<0.05), haptic (F(1, 51) = 11.302; p<0.05) and visuo-haptic (F(1, 51) = 9.82; p<0.05) response modality).

The observed mean reliance on FORs was also compared to the mean reliance on FORs predicred by the Winner-Take-All model. Results are similar to those observed with the MLE rule (i.e., no difference between mean reliance on FORs predicted by WTA model and the observed mean reliance on FORs across response modalities, whereas differences between variance predicted by WTA model and variance observed were significant in the visual (F(1, 51) = 9.594; p<0.05), haptic (F(1, 51) = 8.594; p<0.05) and visuo-haptic (F(1, 51) = 6.976; p<0.05) response modalities.

Ultimately, the observed (mean and variance) reliance on FORs was compared to the predicted (mean and variance) reliance on FORs by the algebraic unweighted mean (UWM) obtained with only one FOR altered (frame tilted or head CM deviated). Results suggest that this model accounts for the observed mean reliance on FORs (no difference between the predicted mean reliance on FORs by this model and the observed mean reliance on FORs across response modalities). Furthermore, this result was also obtained when considering variance (no significant difference between variance predicted by this model and variance observed in all response modalities).

As for sensory information, the present authors also investigated whether the use of the MLE rule to combine FORs was modulated by subjects' degree of reliance on the visual or non-visual FOR. With this in mind, the slope between the mean reliance on FORs predicted by the MLE rule and the observed mean reliance on FORs for each subject was calculated. The obtained individual slopes were then used for correlation analysis with visual or egocentric FOR dependency. The analysis revealed no significant correlation between the slopes and visual FOR dependency or between the slopes and egocentric FOR dependency.

The same analysis was performed with the WTA rule. Analysis revealed no significant correlation between the slopes and visual FOR dependency or between the slopes and egocentric FOR dependency.

**Figure 9:**
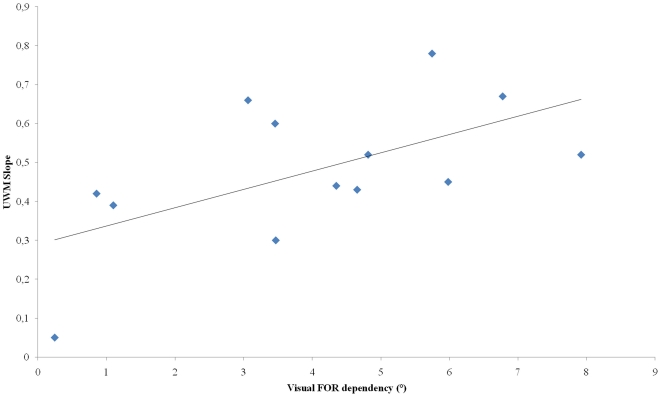
Correlation between individual visual frame effect scores in visual modality of response in the ‘no deviation of head CM’ condition and slope between double disruption response predicted by UWM rule and observed double disruption response.

The same analysis was performed with the UWM *(* *Fig. 9* *)*. Analysis revealed a significant correlation between the slopes and visual FOR dependency (R^2^ = 0.35; p<0.05) but not between the slopes and egocentric FOR dependency. Results show that the more visual FOR dependent the subjects were, the more efficiently the UWM rule predicted the observed mean reliance on FORs in conditions where both FORs were altered (slope close to 1).

## Discussion

### Effects of the tilted visual FOR and CM deviation of the egocentric FOR on SHO:

The results show that visual estimates of head's axis orientation were biased by a tilted visual FOR but not by the deviation of the head's CM. The important inter-individual variability could explain this lack of head CM deviation effect. This is why we conducted individual analyses. The effect of a tilted square frame is a well established finding in SVV [16], [36]–[38] and this effect further extends to body or head orientation [3], [21], [27]. The influence of frame tilt on subjects' estimates indicates that they preferentially align the rod of body axes with respect to axes of the visual FOR. Conversely, the absence of a tilted frame effect on rod settings in some subjects suggests that the rod is preferentially aligned with axes of non visual FOR [1]. Interestingly, the present findings show that the VFE does not systematically extend to rod haptic settings of SHO, suggesting that haptic and kinesthetics cues as well as motor commands likely play a significant role in disambiguating the VFE. This contrasts with earlier results reporting a VFE in active sensorimotor condition in turn providing reliable and unbiased proprioceptive and vestibular cues. Bray et al. [16] showed that challenging postural balance reduced the VFE, without ever cancelling it completely. This reduction is most likely due to the enhanced contribution of non visual cues (proprioceptive, vestibular and motor commands).

These results suggest that the visual frame effect may affect the processing of spatial relationships underlying the control of body orientation except when it involves upper limb control. The present findings are reminiscent of those obtained by [39], [40] who concluded that visual framing had no effect on the execution and endpoint errors in reaching movements.

The less frequent observation of head CM effect on SHO across sensory modalities can be explained by the possibility for subjects to rely on head-trunk articular axes, which remain a reliable and unbiased source of proprioceptive cues throughout the experiment. To verify the assumption that head CM deviations distort the head orientation perception it would be necessary to carry out further studies where proprioceptive cues related to the head or trunk articular axes would be also biased or blurred [41], [42].

We showed that visual and egocentric mass-related FORs dependencies were negatively correlated. This result is consistent with the hypothesis of a hierarchical organization of FORs preferences [43]–[46]. Moreover, subjects displaying the larger frame effect presented the lower head CM effect, further evidencing their preference toward the visual FOR over and above the egocentric FOR. Conversely, subjects with the larger head CM effect presented the lower frame effect, evidencing their preferential use of the egocentric FOR over and above the visual FOR.

Interestingly, the present results showed that biasing the visual FOR (by using frame tilt) not only affected the perception of the head axis orientation (SHO), but this effect was further modulated by the richness of the sensory context wherein the estimations were produced.

### Effects of sensory modalities of response:

The multisensory condition of response (i.e. visuo-haptic) reduced the visual dependency, thus improving SHO. In other words, the haptic orientation cues gathered during the adjustment of the physical rod in combination with its visual displaying on the TV screen allowed subjects to disambiguate the VFE. These results on SHO are consistent and extend to the subjective verticality of those who [21], found that visuo-kinaesthetic settings reduced the effect of a tilted frame (Rod-and-Frame Effect (RFE)). Interestingly, the visuo-haptic reduction of the SHO bias was only observed when the visual FOR was altered. Indeed, the visuo-haptic benefit is never observed in ‘no frame tilt’ conditions. The weak errors observed in no frame conditions, both in visual alone and haptic alone conditions can account for the lack of visuo-haptic benefit in these conditions.

### Optimal rule of combination (MLE) applied to sensory cues and preferential FOR:

The results suggest that visual and haptic orientation inputs are effectively combined to reduce misalignment effect of visual FOR axes on SHO (i.e., visual dependency). The data further shows that the MLE rule seems to account for the observed visuo-haptic improvements. However, it is worth noting that the MLE rule predicts that variance should be reduced in the combined VH modalities of response as compared to V and H taken in isolation. The present results shows that the variance of visuo-haptic estimates predicted by the MLE rule do not fit the scatter of observed visuo-haptic estimates in all conditions combined. Such a conflict was already reported in situations where spatial cues become too incongruent [47], thus leading the combination rule to produce sub-optimal estimates, forcing subjects to rely on one of the other available sensory sources (i.e., a larger variance than expected).

The nature of the instructions given to subjects could also have played an important role. In the present study, subjects were instructed to readjust the rod as precisely as possible, but were not instructed to minimize the scattering of estimations. Further studies will be necessary to address both the precision and accuracy of SHO as well as FOR preference issues.

It was assumed that MLE rule would mainly be used when the congruence between FORs is maximal. Close inspection of the data in each condition revealed that the MLE rule seems to account for the observed visuo-haptic improvements in conditions where FORs congruence is maximal, but also seems to predict improvements observed in almost all other conditions, ranging from intermediate to maximal FORs incongruence. The use of the MLE rule thus appears to be maintained under all circumstances. Similar conclusions may be drawn with respect to the WTA rule (i.e., the use of this rule also seems to be independent of FORs orientation).

The results showed that the unweighted mean and variance of the two modalities of response alone best account for the observed SHO estimates in the VH mode of response. This finding applies when conditions are both combined or considered separately. It may be suggested that when FORs become too incongruent, subjects are not able to rely on one or the other FOR, leading them to solve this issue by trading-off one FOR against the other.

Results showed significant relationships between visual FOR dependency and the use of MLE-type multisensory integration strategies. In highly visual FOR dependent subjects, the MLE rule overestimated actual perception of several sensory cues. Conversely, in less visual FOR dependent subjects, the MLE rule underestimated actual perception of several sensory cues. Similar observation can be made using WTA-type multisensory integration strategies. These results are consistent with the theory of inter-individual differences in that they lead to the recognition of different sensory cues in explaining IDs with regards to visual field. UWM-type multisensory integration strategies seem to be independent from visual FOR dependency.

### Spatial orientation and selection of spatial frames of reference

These results demonstrated the importance of appropriate FOR selection when computing the SHO. Deviation of the visual axes from the head's Z axis alters the precision of rod setting of SHO. These findings emphasize the manner in which spatial relationships were processed by the central nervous system. Furthermore, they are in line with a hypothesis already put forward by some authors, namely that appropriate FOR selection is necessary for producing optimal estimates [3], [48].

Interestingly, the present research has demonstrated that the reliance on visual FOR (field dependency) persists even after subjects' combining of visual and haptic cues. The weighting of the visual FOR for head orientation estimation remains an amazing computational strategy. Indeed, proprioceptive or vestibular systems still provided reliable and appropriate cues with regards to body or head orientation, relative to the support surface or relative to space. The question that remains is why the appropriateness of non visual FORs was not detected for them to subsequently be used to produce more accurate and reliable SHO estimates (persistence of inter-individual differences in VH response modality). The negative correlation between the VFE and CM effect is consistent with the vicarious processes hypothesis [49] involved in the selection of FORs [3], [25], [50]. The vicarious processes would generate these IDs. These spatial idiosyncrasies would reflect stylistic preferences regarding the use of the available FORs, leading to their hierarchical organisation. Within this theory, the level of the task demands would impact this hierarchized use of these FORs, in an adaptive manner toward the use of less habitual FORs, and as consequence to modify the magnitude of these IDs (emergence or disappearance). When situation are not very constraining, different modes of spatial referencing may coexist due to their equiefficiency to control spatial interaction, leading hence large IDs to emerge. Conversely, demanding tasks in reducing the range of adaptive modes of spatial referencing require individual to shift towards the selection of the more appropriate FOR to control spatial interaction efficiently (likely in an optimal manner), leading IDs to disappear. Our study showed that these FORs preferences remained in VH response modality.

The decrease of the frame effect on SHO in visuo-haptic modality of response was observed when the visual FOR was no longer aligned with the head axis orientation. A possible postulate is that in the no frame condition, the discrepancy between SHO and real head orientation is too small to enable a decrease of the SHO bias in visuo-haptic modality of response.

### Optimal rule of sensory combination (MLE) applied to FOR

Results showed significant relationships between visual FOR dependency and the use of UWM-type sensory combination strategies. In highly visual FOR dependent subjects, the UWM rule suitably accounts for the observed mean FOR reliance under conditions of maximal FOR incongruence (frame tilted coupled to deviation of head CM). Indeed, with these subjects, the value of the slope between the mean FOR reliance predicted by the UWM rule and the observed mean FOR reliance is close to 1. In less visual FOR dependent subjects, the smaller slope shows an underestimation of observed mean FORs reliance by the UWM rule. Taken together, large values of visual dependency seem to result from the use of an UWM rule of visual and non-visual FORs.

Taken together, the present results could be interpreted within the framework of the subjective “composite” reference frames hypothesis [21], [51], [52]. This hypothesis would account for the lower than expected errors in subjective vertical (SV), that are induced not only by alteration of various FORs, e.g., tilt of the visual frame [27], [37], [53], but also by body tilt [54], modifications of the gravitational field [55] or even the alteration of trunk mass distribution [1]. With this view, and in line with the present results, SHO is neither perfectly lined up with the egocentric FOR, nor with the visual FOR. Results can thus be interpreted as the consequence of multiple influences of the different FORs. The CNS may reinterpret the different FORs to create a new subjective “composite” FOR. This new subjective “composite” FOR is created by assigning a weight to each FOR as a function of task constraint. In other terms, the more unreliable a FOR, the heavier the weight ascribed to other FORs. This assumption is consistent with work by Howard (1982, 1986) [56], [57], who showed that different FORs contribute to the cognitive determination of the SV. However, McGuire and Sabes [58] provided proof against the hypotheses according to which the pattern of perceptual errors would reflect differential reliance on either a specific FOR, a common FOR, or a hybrid FOR. The above authors proposed that perception is simultaneously specified in multiple FORs and that their respective statistical reliability influences their relative weighting. The effect of visual frame on SHO observed in the present experiment could reflect simultaneous influences of different FORs and their alternation. Bistable perception was recently evidenced between auditory and haptic cues as well as between two different olfactory stimuli presented to each nostril.

### Spatial orientation and neural basis of FOR selection and change

Deneve and Pouget [48] proposed two hypotheses to account for neural mechanisms' implementation of a cross-modal spatial link, stating the following: “sensory remapping, which would involve the recoding of all sensory inputs in a common frame of reference on a multisensory brain area, and direct cross-modal influence, whereby sensory activity in one unimodal brain area directly influences sensory activities in another unimodal area” (p. 253). Several studies supporting both hypotheses, authors suggest the existence of “a role of both feed-forward connections from unimodal to multimodal areas and feedback connections from multimodal to unimodal areas”. Avillac and al. [59] investigated the combination of visual and tactile inputs in macaque monkeys, obtaining results consistent with a model of multisensory integration based on multidirectional sensory predictions. Neurophysiological studies provided evidence of a sensory weighting and reweighting mechanism, originating in functional inter-sensory reciprocal inhibitory interactions (RII) underlying sensory cortical brain activation and deactivation observed during competing visual and vestibular inputs [60]–[62]. These RII are likely to work regardless of the sensory channels involved. Nevertheless, the strength of these inter-sensory RII for reweighting cues and hence in reducing sensory mismatch is likely to depend on prior experiences, that are known to shape well-defined and well-structured somesthetic maps [63]. Resistance to misleading visual FOR should thus depend on subjects' sensorimotor experiences, which, by improving the definition and structure of egocentric somato-proprioceptive maps [64], [65] would enhance their capability to accurately perceive the proprioceptive orientation of their limbs. Several authors have suggested that the reliance on visual FOR could be due to difficulties in using proprioceptive or vestibular cues to check FOR appropriateness as well as elicit FOR changes when necessary [3].

### Conclusion

This study analyzes i) the relative contribution of visual and egocentric frames of reference in the subjective head orientation perception (SHO) and ii) the relative contribution of several sensory cues in subjective head orientation processing. The main results are that disrupting the visual reference frame, in turn, disrupted subjective head orientation. A disruption of the egocentric frame of reference, however, did not. Nevertheless, these results are modulated by individual analyses; some subjects are more affected by the alteration of the visual frame of reference, whereas others ones are more affected through altering the egocentric frame of reference. The negative correlation observed between visual and egocentric mass-related frames of reference dependencies are consistent with the hypothesis of a hierarchical organization of frames of reference preferences. Another important result is the reduction of visual dependency in the multisensory condition of response (i.e. visuo-haptic). The haptic orientation cues gathered during the adjustment of the physical rod in combination with its visual displaying on the TV screen allowed subjects to disambiguate the visual frame effect. Finally, relationships between visual FOR dependency and the use of UWM-type sensory combination strategies show that highly visual FOR dependent subjects seem unable to rely on the more appropriate frame of reference (i.e. the less biased), and minimize the influence of “wrong” frame of reference by using an unweighted rule of available frames of reference.
